# ‘Somewhere between science and superstition’: Religious outrage, horrific science, and *The Exorcist* (1973)

**DOI:** 10.1177/09526951211004465

**Published:** 2021-05-10

**Authors:** Amy C. Chambers

**Affiliations:** Manchester Metropolitan University, UK

**Keywords:** psychiatry, science and cinema, science and religion, science communication, *The Exorcist* (1973)

## Abstract

Science and religion pervade the 1973 horror *The Exorcist* (1973), and the film exists, as the movie’s tagline suggests, ‘somewhere between science and superstition’. Archival materials show the depth of research conducted by writer/director William Friedkin in his commitment to presenting and exploring emerging scientific procedures and accurate Catholic ritual. Where clinical and barbaric science fails, faith and ritual save the possessed child Reagan MacNeil (Linda Blair) from her demons. *The Exorcist* created media frenzy in 1973, with increased reports in the popular press of demon possessions, audience members convulsing and vomiting at screenings, and apparent religious and specifically Catholic moral outrage. However, the official Catholic response to *The Exorcist* was not as reactionary as the press claimed. The United States Conference of Catholic Bishops’ Office of Film and Broadcasting (USCCB-OFB) officially and publicly condemned the film as being unsuitable for a wide audience, but reviews produced for the office by priests and lay Catholics and correspondence between the Vatican and the USCCB-OFB show that the church at least notionally interpreted it as a positive response to the power of faith. Warner Bros. Studios, however, were keen to promote stories of religious outrage to boost sales and news coverage – a marketing strategy that actively contradicted Friedkin’s respectful and collaborative approach to working with both religious communities and medical professionals. Reports of Catholic outrage were a means of promoting *The Exorcist* rather than an accurate reflection of the Catholic Church’s nuanced response to the film and its scientific and religious content.

One of the best things that could happen is if the Pope denounces it.

- William Friedkin, director of *The Exorcist^1^*

*The Exorcist* appears to have everything – including Catholic approval.

- Rev. Lester Kinsolving (1974), Episcopalian newspaper columnist

The entanglement of Catholicism with psychiatry, science, and medicine possessed the popular American imagination in the 1970s. This fascination was crystalised in William Friedkin’s iconic film *The Exorcist*, released in the United States in December 1973, in which an innocent young girl is seemingly demonically possessed and subjected to tests to decipher where specifically her affliction sits in terms of the realms of science and superstition. *The Exorcist* became a long-term cultural touchstone for discussions of the tension between science and religion, the representation and treatment of mental health, and the position of religion in contemporary North America.

The Protestant magazine *Christian Century* ran an article connecting *The Exorcist* with the Watergate scandal, referring to the two events as ‘psychodramas of the American soul’.^2^ Responses like this were frequent and articulated fears of a church seemingly losing its control over American society and its soul, whilst simultaneously highlighting the prevalence of the psy-sciences in US popular discourse. Protestant commentator Carl Raschke claimed that the Devil was having ‘his day’ and that the film reflected ‘the cynical mood of our age [arising] by default from the wreck of traditional religious as well as social values’.^3^ Thus, *The Exorcist* is positioned at the intersection of a hugely volatile and disruptive moment in US history. Although *The Exorcist* was part of a series of popular culture texts to reflect upon these ideas, it did so with such a level of compelling scientific and religious fidelity that it should be understood as cultural moment that engaged with the social and cultural issues of the long 1960s – an era where Americans were questioning many of their central authorities and institutions.

*The Exorcist* connects the worlds of science and religion through their individual responses to the seen and unseen, and the known and unknown. For science, these apparent binaries are at the centre of research across discovery, observation, and critical and peer review of findings in attempts to ensure ‘a special kind of reliability’ (Chalmers, 1999: ixx). *The Exorcist* offers a critique of what might be considered ‘the objectifying tendency of science’ (Crawford, 1998: 36). The increasing commodification and mechanisation of medicine delegates decisions about medical treatment and ethics to modern medical technologies. Yet this is not taking place in ‘some abstract, science fictional future’ or in horrific cinematic imagination; it is happening in the present (Wald, 2012: 202). The medical and psychiatric space (the hospital and the clinic) takes on a different role – that of the space not of logic, cure, and control but of trial and (more than often) error, as these professionals are unable to diagnose their patient. The most visibly horrific scenes, as pertaining to the horror film genre, occur in the operating theatre and not in the realms and representation of the supernatural.

Medico-psy-scientific and religious authority was under public scrutiny during the period surrounding *The Exorcist*’s release: ‘Ordinary Americans were overhauling how they understood cultural authority…and how professional experts represented reality itself’ (Quinlan, 2014: 328). Doctors, scientists, and priests, as traditional authority figures, ‘appeared potentially self-interested or ineffective, proffering biased knowledge under the guise of scientific-institutional objectivity’ (ibid.). They seemingly failed to adequately explain or eradicate the national and international traumas of the 1960s and 1970s, including the Vietnam War, Watergate, and the dismantling of traditions and intolerances by the counterculture, including civil rights, women’s rights, and the emergence of ‘alternative’ approaches to science and religion. In a blending of ‘the conventional and the countercultural’, young people were not rejecting science and religion entirely but rather seeking experiences beyond the state-sponsored ‘big science’ that had defined the US post-1945, and more personal transcendent experiences inspired by ‘Eastern religions and chemically enhanced spirituality’ (Kaiser and McCray, 2016: 2). Films of the era, including *A Clockwork Orange* (1971), *The Exorcist*, *One Flew Over the Cuckoo’s Nest* (1975), *Halloween* (1978), and *When a Stranger Calls* (1979), ‘articulated long-standing American fears of tyranny, control, and gendered disorder’ (Rondinone, 2020: 902). These films, as Troy Rondinone (ibid.: 925) argues, can be used to ‘trace the trip from distrust to institutional annihilation’, specifically of US mental health facilities, but more broadly of the major US institutions of power, including government, church, business, and the sciences.

As a reflection of and an engagement with the issues of the era, *The Exorcist* was inevitably permeated with discussion and critique of science and religion. The film exists, as the movie’s tagline suggests, ‘somewhere between science and superstition’, but it is not simply a battle between the two but rather a ‘critique of strict allegiance to a set of extremes, whether “good or evil” or “faith or science”’ (Dudenhoeffer, 2010: 76). *The Exorcist* was also released in the aftermath of the Second Ecumenical Council of the Vatican (1962–5), which addressed the relationship between the Catholic Church and the modern world. Vatican II, as it is commonly known, was announced in 1959 with three purposes: ‘the spiritual and pastoral renewal of the Catholic Church, the updating of the Church’s outlook and institutions…, and ecumenical reconciliation with non-Catholic Christians’ (Marshall, 2017: 999). The church wanted to be seen as a modern institution that was not in conflict with science, shown through a willing acceptance of ‘sociology, psychology, and psychoanalysis’ over the concept of evil for explaining humanity’s problems (Jancovich, 1992: 93–4). This more liberal approach to the sciences is imitated in *The Exorcist* through the scientist-priest characters, Fathers Karras (Jason Miller) and Merrin (Max von Sydow).

*The Exorcist* is adapted from William Blatty’s novel *The Exorcist* (1971) and follows the experiences of 12-year-old girl Regan MacNeil (Linda Blair) and her actress mother Chris (Ellen Burstyn) as they take up temporary residence in Georgetown in Washington, DC for an on-location movie shoot. Regan begins to behave strangely, and then profanely and violently. Chris seeks medical attention, and several visceral invasive medical procedures are used to attempt to diagnose somatic causes, but Regan’s apparent illness appears to be beyond the expertise and experiences of hospital doctors and psychiatrists. What Regan needs is an exorcist. Chris meets with Father Karras (Jason Miller), a Roman Catholic priest and psychiatrist who is losing his faith and dealing with a terminally ill mother, whose medical care he cannot afford. As it becomes apparent to Karras that Regan is possessed and an appropriate candidate for the ancient ritual of exorcism, he requisitions the services of Father Merrin (Max von Sydow), an elderly priest and archaeologist who has just returned from Iraq with premonitions of coming evil. The demon, or perhaps the Devil itself, is exorcised from Regan at the cost of both priests’ lives: Merrin dies from a heart attack and Karras is willingly possessed by the demon, killing himself in a moment of faith and sacrifice. Where a clinical and at times seemingly barbaric adherence to science fails, ancient ritual and the power of restored faith save the possessed child from her demons.

*The Exorcist* was originally released on 26 December 1973 in the Christmas period (Thanksgiving to New Year) of the US blockbuster calendar (Neale, 2003: 55). This release date was considered inappropriate by the Catholic Church but was seen as one that, as one priest in correspondence with Friedkin explained, ‘ought to get the crowds [because] weary shoppers are always calling upon the devil and wishing others to reside in hell at that time’.^4^ International release dates were spread across 1974 from the March of that year into early 1975. The film caused controversy wherever it went because of its salacious and blasphemous religious content, including scenes of the possessed child masturbating with a crucifix, and this controversy was exacerbated by increasing reports of requests for exorcisms and strange behaviour at cinema viewings.

The intensity of *The Exorcist*’s cultural impact ‘stretched far beyond its power to overwhelm audiences’ (Poole, 2009: 156). As W. Scott Poole argues, in the late 1960s into the 1970s, there was ‘a moment in American cultural life when the Devil occupied a place in the public discourse’, serving as a character and a cumulative site for a variety of cultural narratives of the 1970s, often including mistrust in the institutions of both science and religion (ibid.). *The Exorcist* tapped into the fears of the zeitgeist, including invasion of society by the other (individuals, groups, or ideologies) and the disruption of the morals, minds, and bodies of America’s future. This corruption is often represented by possessed children in the Satanic movies of the era, because they are ‘the carriers for the group’s and species’ genetic future’ (Fry, 2015: 19). *The Exorcist* became a reference point for decades to come – with its continued receivership ensured by its controversies and urban legends – in the discussion of uncontrollable youth, the psy-sciences, and the positioning of religion in the long 1960s. ‘The reality of history deconstructed the deeply held belief in American innocence’ that had emerged and been entrenched by the 1950s but had become increasingly difficult to uphold following the civil rights movements and anti-war protests of the 1960s (Poole, 2009: 156). As an exemplary film of the era, *The Exorcist* communicates an image of a United States in an unstable state of change that can no longer avoid its real and historical systemic evils.

Recently released archival materials show the depth of research conducted by writer and director William Friedkin in his commitment to presenting and exploring emerging scientific procedures alongside accurate Catholic ritual.^5^ *The Exorcist* created a media circus in December 1973 and throughout 1974, with increased reports in the popular press of demon possessions, audience members convulsing and vomiting at screenings, and apparent religious and specifically Catholic moral outrage. The official Catholic response to *The Exorcist*, however, was not as reactionary as the press claimed. The United States Conference of Catholic Bishops’ Office of Film and Broadcasting (USCCB-OFB) officially condemned the film as being unsuitable for a wide audience.^6^ But reviews produced privately for the office by priests and lay Catholics, and correspondence between the Vatican and the USCCB-OFB, show that the church at least notionally interpreted it as a positive response to the power of faith. Warner Bros. Studios, however, were keen to promote stories of religious outrage to boost sales and news coverage – a marketing strategy that actively contradicted Friedkin’s respectful and collaborative approach to working with both religious communities and medical professionals. Reports of Catholic outrage were a means of promoting *The Exorcist* rather than an accurate reflection of the Catholic Church’s nuanced response to the film and its scientific and religious content.

## Science, religion, and post-classical Hollywood

From around 1930 to 1968, most films released by Hollywood studios were censored in some way. This was an era of censorship that was in part the result of Christian campaigns as well as of industry fears of direct government intervention. At the end of 1960s, the US Catholic Church was emerging from the cessation of a long period of notable cultural control as movie censors after over 30 years of direct influence. The authority that the US Catholic Church felt it had over US society and its values as expressed in the Hollywood movie was diminished from censor to commentator.

During the late 1920s and into the 1930s, several Christian denominations were involved in the popularisation and ultimate acceptance by Hollywood studios of a moral code of production that aligned with Christian values (Black, 2013; Leff and Simmons, 1990). The Motion Picture Production Code (1930–68) allowed censors and religious groups – the Production Code Administration (PCA) and the National Legion of Decency – to request changes to film content in pre- and post-production. Studios were required to submit treatments and screenplays for approval and make changes to shooting scripts in accordance with the advice given, and then submit the final cut of the film for consideration, which would often result in edits and reshoots. In order for a film to be given general release and considered appropriate for all audiences, film-makers had to work with censors and religious commentators.

Religious groups and predominately mainline Christians have ‘often attempted to influence the way stories about science have appeared on cinema screens’, crediting the movies with the power to influence and even corrupt their viewers (Kirby and Chambers, 2018: 279). Between the 1930s and the 1960s, religious censors had the power to request changes to Hollywood movie science that they found incompatible with their faith, such as Christian notions of the human mind, body, and soul. They could control to an extent which stories were being told about science and how audiences would receive them. The Production Code lost much of its power by the 1960s due to broader cultural changes, including the rise of television, an increasingly permissive social stance towards sexual matters, and a more socially progressive attitude in the Catholic Church. The end of this era of censorship saw a shift in the intersection of science, religion, and cinema, as Christians lost their direct control over movie content.

Despite the relaxation of the US Catholic Church’s official attitudes to on-screen depictions of science, concerns amongst religious groups about scientific content in films remained after the end of official censorship. Without the power to censor movies, however, these groups had to find other ways to influence the way audiences interpreted cinematic stories about science. They were engaging with science narratives rather than altering or preventing them, and began producing and disseminating ratings, reviews, and educational viewing guides and organising boycotts and picket lines. These interceptions at the point of reception were not the only way that the Catholic Church gained influence; the popularity and wide dissemination of the *Catholic Film Newsletter* and its extensive reviewing of films on general release did lead to some film-makers choosing to consult with the church and voluntarily offering changes in their content in order to gain a favourable rating that would affect audience numbers in religious communities (Kirby and Chambers, 2018).

The introduction of a ratings system and the resultant shift to the Catholic Church acting as a critic rather than a censor signalled an apparent loosening of control *and* public morals – a moral decline that coincided with the release of ‘a Catholic horror film [and] more specifically…a Jesuit horror film’ called *The Exorcist* (McDannell, 2008: 199). *The Exorcist* quickly became a cultural touchstone that would thereafter be associated with the church. The film acted as a bellwether for the New Hollywood that would arise, as ratings replaced censored and essentially Catholic-approved and mediated content. ‘Boycotts, picketing, and “C” ratings had not staunched the flow of ever more daring Hollywood films’ throughout the 1960s (Skinner, 1993: 153). In 1965, the National Legion of Decency’s name was changed to the National Catholic Office for Motion Pictures (NCOMP), and the dreaded C rating was replaced with the more ambiguous A-IV rating.^7^ These changes were a ‘concession to shifting mores’ and indicated that the church had accepted that in order to remain relevant, it would need to adapt its public-facing approach to movies (ibid.: 154).

The new freedom given to film-makers by the industry’s rating system allowed for movies that ‘positioned controversial science and scientific ideas at the core of their narratives’ (Kirby and Chambers, 2018). *The Exorcist* could show bloody and painful medical procedures *and* demonstrably negative or satirical images of the Catholic Church that would have been strictly prohibited by the PCA (Leff and Simmons, 1990). *The Exorcist* was also part of a series of post-classical Hollywood films that relied on Catholic storytelling and themes: the lapsed Catholic faith in *Rosemary’s Baby* (1968), the depravity in *The Devils* (1971), the hypocrisy of *The Godfather* (1972), the urban Catholicism of *Rocky* (1976), and the violence of the Devil in *The Omen* (1976). New rules made New Hollywood, and the end of restrictions against scientific images and the critique of religion opened up opportunities for film-makers as well as new challenges for US Catholics and members of other Christian denominations (including Lutherans and Protestants) who sought to ‘control’ the interpretation of such cinematic ideas.

## *The Exorcist*: Exploitations, exaggerations, and exorcisms

**Figure 1.**
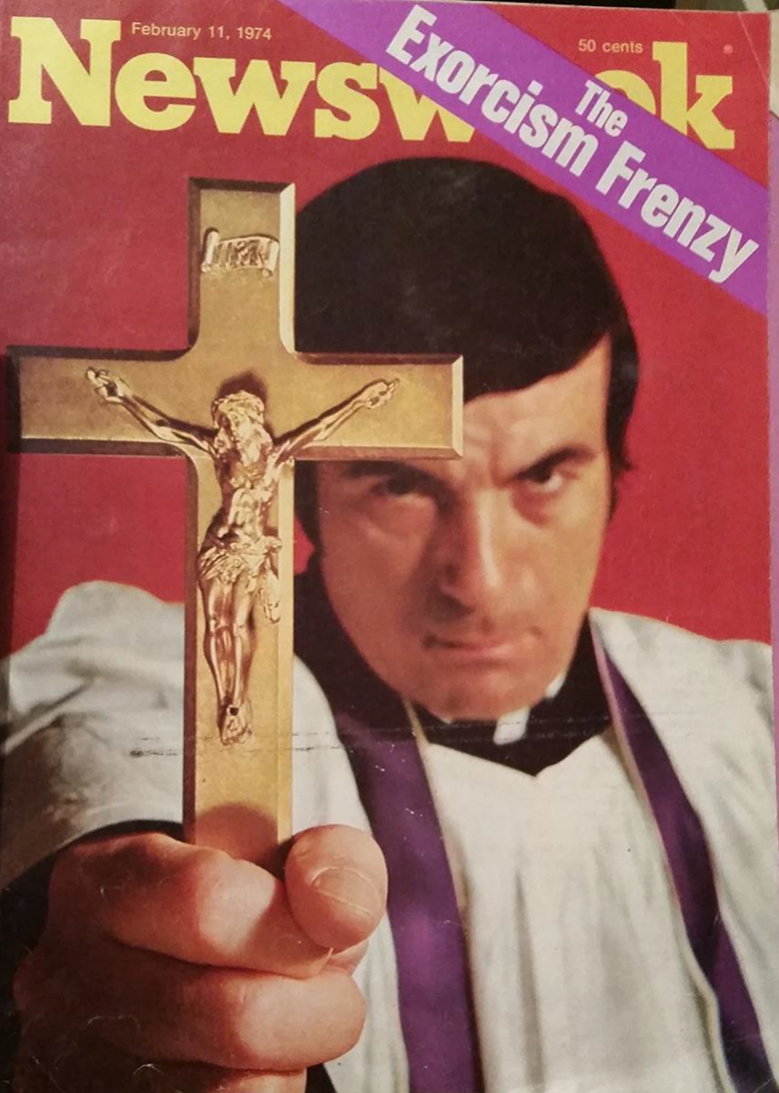
Front cover of *Newsweek* (11 February 1974).

The 1973 Christmas release of *The Exorcist* was accompanied by a media frenzy; the most extreme reports conveyed patrons going into cardiac arrest and spontaneous abortion (Kermode, 1998: 84). These were accompanied by more frequent stories of cinemagoers fainting, vomiting, and shouting during screenings, and of increased reports of breakdowns, suicide, and possession in the weeks after seeing the film. The North American weekly *Newsweek* (Figure 1), in its 11 February 1974 edition, ran a cover story titled ‘The Exorcism Frenzy’ (Woodward, 1974: 60–6). It featured stories about janitors up to their ankles in vomit in cinemas in Illinois, a Californian moviegoer charging at the screen to ‘get the demon’, Boston Catholic Centers receiving daily requests for exorcisms, and psychiatrists’ opinion pieces mooting the idea that possession was ‘not inconceivable’. These stories were underpinned with narratives about the moral outrage of the Catholic Church and highlighted the cautionary classification given to the film by the Catholic Office of Film and Broadcasting in the *Catholic Film Newsletter*.

The official review published in the *Catholic Film Newsletter* on 15 January 1974 gave the film a rating of A-IV, with quite a conservative and negative write-up. This response was officially built from the responses of the priests and lay Catholics who had seen and commented on the film, but it also seemed to reflect wider concerns about how the film would inevitably be aligned with the church.^8^ The collated review was quick to highlight the rarity of exorcism in the Catholic Church and the fact that ‘modern knowledge of psychosomatic disorders [explained] why the Church in recent times has rarely approved the use of the rite of exorcism’ (NCOMP, 1974). The piece also suggested that the film was a reflection of ‘sick faddist trends in contemporary society’ and ‘fascination with the occult and devil worship’, with the film being at risk of confirming rather than rejecting such blasphemous approaches – especially since the film ended in a particularly problematic way for Catholics, as ‘either the devil kills the priest or he, in an act of heroism, commits suicide’ (ibid.). Other Christian publications and commentators used the film to critique the Catholic Church, the USCCB-OFB, and the A-IV rating (which was often incorrectly reported as a C rating in the popular press), and the USCCB’s cooperation with the film-makers.

Drawing upon the rhetoric surrounding *The Exorcist* in the popular and religious press, the ecumenical (Protestant) Christian weekly *The Courier* ran the headline ‘Satan Goes to the Movies’ alongside their coverage of the film and its controversies (Carter, 1974: 5). Interviews appeared with key figures, which seemingly gave them the opportunity to defend their inclusion. Father John J. Nicola, SJ, who served as a credited advisor for the movie, was frequently quoted and profiled, but he often alerted journalists to the fact that he had ‘staged his own small protest’ by resigning from the project as an actor; he was originally cast as the president of Georgetown University, a role later played by Father Thomas Bermingham, SJ (Gildea, 1974). Father Nicola’s refusal to literally play a part in *The Exorcist* followed disagreements with William Friedkin over the inclusion of the simulated desecration of the Madonna at Holy Trinity Catholic Church (Georgetown) and the infamous crucifix masturbation scene (ibid.).^9^ Even those who were directly involved in the project and suggested positive responses to the film were still eager to keep their distance.

Protestant revivalist the Rev. Billy Graham, who was a full-time evangelist of the Youth for Christ organisation, condemned *The Exorcist* by remarking that watching the film was akin to exposing oneself ‘to the devil’ (Graham, 1974) and claimed that the film was ‘a sort of spiritual pornography, pandering to man’s innate superstition’ (Cox, 1974: 10). His opinions on the film aligned with his own history of undermining the Roman Catholic Church and the exorcism ritual, claiming that it was ineffective against the Devil. Here, *The Exorcist* became a neat touchstone in the discussion of the evils of society across several institutions, but most often the Catholic Church and the politics and procedures of medical facilities. In his widely syndicated weekly ‘Inside Religion’ column, Episcopalian priest the Rev. Lester Kinsolving (1974) argued that ‘*The Exorcist* appears to have everything – including Catholic approval’ and incorrectly reported that the film had been classified as A-III (suitable for adults).^10^ In an ‘editorial departure from its usual ecumenical stance’ (Woodward, 1974: 61), *The Christian Century* denounced *The Exorcist* as a ‘hard-core pornography’, opining that ‘by our protestant standards [*The Exorcist* offers] a completely impossible solution’ to the real possibility of evil. *Newsweek* reported that in Washington, DC there were ‘waiting teams of Methodist evangels passing out leaflets inviting standees to decide whether “you will be controlled by the spirit of darkness or by the spirit of God”’ (ibid.: 61–3). Numerous local and national predominately Protestant groups organised boycotts and campaigns outside cinemas to warn people about the evil of the film, and in some cases, groups offered support to audience members after seeing the film, providing local parishioners’ phone numbers and details of support meetings. Although the Catholic Church was said to have contributed to a film that was tantamount to a ‘recruiting poster’ (Kael, 1974: 60), the Protestant denominations utilised *The Exorcist* to criticise the Catholic Church, highlight their own piety and ‘correct’ understanding of the Christian faith, and promote Protestant publications and pews.

Newspapers were fascinated with how the audience was physically and mentally affected by ‘vicariously experiencing super-natural events’ (Fiske, 1974; Heisler, 1975). There were reports in local, national, and syndicated press about apparent resultant increases in church numbers (Ho and Wermiel, 1974; Keegan, 1974), requests for exorcisms (Page, 1974), and visits to medical professionals (Greenson, 1974). The furore surrounding *The Exorcist* essentially marketed the film for the studio and distributors (both domestic and international), and as Pauline Kael (1974: 60) adroitly noted in her *New Yorker* review, ‘complainers became accessories’ to the film’s success.^11^ The more religious groups, whether Catholic or not, increasingly pushed back against the film and its content the higher the box office figures rose, which also led to greater coverage in the press at home and abroad in advance of the international release in March 1974. Warner Bros. were delighted by the stories of religious outrage, as they correlated the news coverage with boosted sales – with internal memos remarking that the best thing that could happen would be a public condemnation from the Pope. However, this was a marketing strategy that did not align with Friedkin’s attitude to working with both the Catholic Church and medical professionals.

## Systems of science: Diagnosis/cure

The purpose of interpolating fresh scientific material into *The Exorcist* was, as director William Friedkin explained, to ‘root the picture in time, recent time’ and as far as possible to enable modern audiences to have realistic and rational perceptions of what was happening to the seemingly innocent Regan.^12^ In an internal Warner Bros. memo, Friedkin explained that he and Blatty wanted to include what was ‘most up to date’ in the film:

If we were making the picture a year from now, there’d be even greater advances in diagnosis and treatment to the brain. The more you diagnose and are able to treat the brain, the less one relies on exorcism.^13^

As researcher P. B. Ross remarked in the same memo, they actively explored ‘provocative branches of medicine, psychsurgey [sic], arteriography, [and] pneumo-encephalography’.^14^ Friedkin and Blatty’s approach to science and religion suggested that Regan’s unexplained behaviour was unfixed, and that *The Exorcist* reflected the scientific knowledge of the time. If new technologies and treatments had been available, which they claim they would have included, perhaps the movie’s outcome and possible interpretations might have been different.

**Figure 2.**
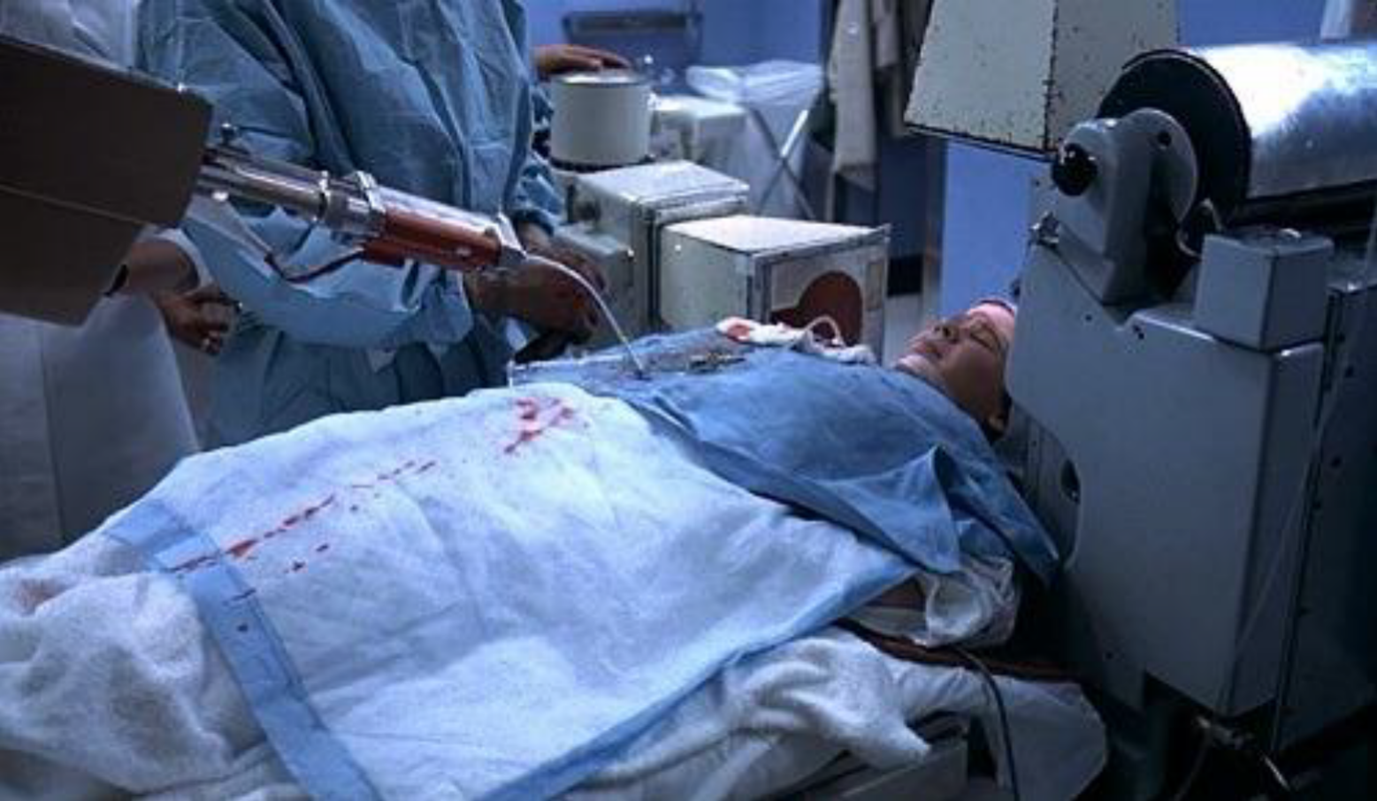
Regan’s (Linda Blair) pneumoencephalogram in *The Exorcist*. On location at Bellevue Hospital (New York) with doctors and radiologists working at the hospital in August 1972.

Science, and specifically medicine, is presented as being unstable and inconclusive but evolving, with the underlying suggestion that it was only a matter of time before these unexplained phenomena would receive a reasonable *scientific* explanation. *The Exorcist* shows the processes of science: the frustration of failure and the reality of using new and old mechanical medical technologies. It demonstrates the objective reality of the practice whilst also raising more subjective social and ethical issues surrounding medical research and testing. Regan is framed as an experimental subject to be controlled, probed, dissected, and resolved. Regan is extensively cannulated and scanned in attempts to uncover hidden intracranial lesion or blood clotting. She undergoes a cerebral angiography by direct carotid arterial puncture, wherein dye is injected in order to make the structure of the brain easier to view on an X-ray, and then the more extreme but older procedure of pneumoencephalography (PEG), which was phased out soon after the film was released (Figure 2). *The Exorcist* offers the ‘most notable depiction’ of PEG and ‘provides us [neurosurgeons] with the most readily accessible historical documentation of the procedure’ (Ishaque, Wallace, and Grandhi, 2017: 6). The processes are shown to be painful, and then uncomfortable, clinical, noisy, and ultimately inconclusive.

A review in the British-based *New Scientist* magazine by comedian and trained physician Graeme Garden argued that the medical scenes were ‘irresponsible’:

The really irresponsible feature of this film is its presentation of the medical sequences…with squirting carotid blood, screaming child and mother, and badly oiled x-ray equipment which screeches and crashes with a sound of a trip hammer exterminating old trams. A genuinely disturbing scene, hardly likely to be welcomed by neurologists. (Garden, 1974: 38)^15^

The medical scenes were the cause of much of the distress in the cinemas, rather than the images of the occult. In 1988, William Blatty gave an interview to film journalist Nat Segaloff where he irately rejected claims of such physical responses to the film, saying, ‘All this stuff about people vomiting – it’s nonsense’ and that it was always the scene with the lumbar needle and not the exorcism that led to walk-outs.^16^ As he explained, ‘That’s the point at which *everybody* got ill and which I always have to hang my head’.^17^ Although it was religious content and ‘frenzy’ that got the majority of the press attention, it was the medical sequences that affected audiences the most. Friedkin’s attention to medical accuracy is where many of the horror components of the film are founded; the exorcism was intense but the medical sequences actually scared people out of their seats.

The medical space is made spectacular and horrific in *The Exorcist* not through the presentation of Regan’s behaviour or the demon, but through the clinical nature of her treatment and how this is communicated through shots and sound.^18^ As Ilkka Mäyrä (1999: 159) notes, ‘The violent movements and noises of arteriographic machinery reach diabolical dimensions’ and the Latin medical terms for possible medication ‘gain occult resonances: *Ritalin, Librium*’. Behind-the-scenes photography and footage for *The Exorcist* show William Friedkin playfully entertaining Linda Blair (Regan) as she is dressed in hospital robes with a milkshake in hand; the scenes that appear in the movie place Regan under a clinically medical and cinematic gaze. Scenes with doctors and nurses taking blood and conducting non-invasive tests are softened with questions about how Regan is doing. In contrast, technical and medical detail take centre stage over the patient in the angiography and PEG sequence (Figure 2): intimate close-ups and medium close-ups stress Regan’s discomfort, with establishing shots highlighting Regan’s smallness in comparison to the large, loud machinery.

Once the possible physical somatic causes of Regan’s affliction are exhausted, the medics turn to the psy-sciences. Regan is sent to the Barringer Clinic, which Troy Rondinone (2020: 905) identifies as ‘an apparent riff on the famed psychiatric facility the Menninger Clinic’. The scene in the clinic further visualises Regan’s dislocation and distance from a diagnosis, let alone a treatment or cure. Regan is projected into the Barringer Clinic scene as a spectre on a grainy closed-circuit television screen as her mother Chris faces a wall of white-coated doctors. Chris is analysed by the director and his staff as her religious beliefs are interrogated. Chris’ response that she is atheist and that her daughter has not been raised in a faith further highlights her frustration. Regan is then presented as a possible case of clinical cacodemonomania, where the patient believes they are possessed – a delusion that might be ‘treated’ through the ‘stylized ritual’ of exorcism.

Research conducted in pre-production included meetings with medical practitioners and visits to hospitals and labs. Friedkin created extensive diagrams and notes about the internal workings of the body and how advances in medical technology (particularly neuroimaging^19^) would increase the likelihood of diagnosing brain tumours and other diseases of the central nervous system.^20^ Friedkin consulted with doctors working at the New York University Medical Center, Colombia Presbyterian Hospital, and Georgetown Medical Center – the ‘three of the best hospitals for X-ray of the brain and psychosurgery…[with] the leading men in the field’ – and used the facilities of Goldwater Memorial Hospital and Bellevue Hospital in Manhattan for on-location shoots.^21^ He discussed emergent technologies as well as those currently available in most hospitals with the doctors he consulted. Extensive notes were made on draft scripts, including three sides of handwritten notes in Blatty’s editor’s script from April 1972 concerning filming and editing in clinical techniques and instruments. Science advisors included psychologists, psychiatrists, neuroscientists, hospital clinicians, and interventional radiologists, who provided step-by-step instructions for depicting the procedures. Friedkin creates a ‘striking medical realism’ by including graphic shots of medical practices that had not been shown on screen before and detailed images of the brain following extensive X-rays (Quinlan, 2014: 323). Friedkin (2012) later claimed that *The Exorcist*’s carotid arteriogram and X-ray imaging footage was used for training radiologists due to his attention to accuracy in these scenes.

The history of cinema is ‘bound up with medical cinematography’ and the processes of recording new ways of knowing and visualising a world that is both seen and unseen, known and unknown (Crawford, 1998: 24). *The Exorcist* was the first time that such graphic medical scenes and detailed clinical images were made easily available to the general public in mainstream cinema. The audience are not simply being told about medical and psychiatric treatments and then being asked to believe the conclusion. Instead, they are asked to *believe their own eyes*, as evidence is sifted and presented with a visual accuracy that reflects the director’s attention to technical detail and the film industry’s changing responses to on-screen representations of science *and* religion.

*The Exorcist* shows ‘science in action’, where the instruments and medical technologies become ‘crucial elements’ to the understanding of the science and the story rather than mere theatrical settings and props (Latour, 1987: 69). As the rules and expectations surrounding Hollywood changed in 1970s, graphic medical imagery became more frequent as a site of both knowledge and body horror. Graeme Garden’s *New Scientist* review claimed that the medical scenes were irresponsible and unnecessarily graphic, but they provided a scientific realism that film-makers had rarely presented, or had been censured from presenting to audiences, prior to this moment in cinematic history. The doctors fail to diagnose Regan, but this is achieved through clinical and methodical experiments – *The Exorcist* does not so much question science as present it as an incomplete and evolving process. *The Exorcist* shows medical technology in use with the reality of the discomfort and noise, rather than presenting it with depersonalised resultant images and conclusive diagnoses; the processes of science are shown rather than just being assumed, and the research into the medical is as vital as the collaboration with the US Catholic Church.

## Between science and superstition

William Friedkin was committed to exploring emerging scientific procedures alongside presenting accurate Catholic ritual. Both sides of the story were researched to a similar degree, ensuring that representations of both scientific method and religious ritual were correct at the time of the film’s release. Whilst contemporary audiences might not even flinch at the visceral arteriogram, the original audience were seeing one on screen for the first time. The inclusion of these images of medical technology was intended to give the medical scenes a futuristic edge to heighten the contrast with the ancient ritual.

Friedkin was considerate of the Catholic Church with the USCCB-OFB, who acted as a point of contact with the Catholic Church in the United States and the Vatican. Friedkin remained in contact with key figures at the USCCB-OFB throughout the process of adapting and shooting William Blatty’s novel; he liaised with bishops and priests in Georgetown, where *The Exorcist* is set, and more broadly with the Roman Catholic Archdiocese of Washington.^22^ There were detailed discussions of the use of church locations and whether scenes should be recreated on studio lots rather than on hallowed church grounds. Friedkin, in his commitment to authenticity and accuracy, wanted to shoot the film entirely on location but was denied access especially for the scene with the mutilated ‘Statue of Our Lady’ at the Holy Trinity Catholic Church.

The balance and indeed ‘tension’ at the centre of *The Exorcist*’s film adaptation is between ‘the religious or mythical’ and a demand for realism, authenticity, and scientific believability (Mäyrä, 1999: 144). When Blatty’s novel was optioned for adaptation, he asserted that the film should have the ‘look of documentary realism’ (Travers and Reiff, 1974: 28), which aligned with the narrative he spun around the novel, in which he claimed that he did not ‘consciously [formulate] the plot for *The Exorcist*’ and that his main contribution was ‘researching the symptomology of possession and the medical information’ (Blatty, quoted in ibid.: 16). Sara Williams (2011) argues that the ‘complexity’ of the novel is often overlooked and perhaps overshadowed by the film, which offers a far more conservative view due in part to the ‘abandonment of the [novel’s] psychological realism’ concerning Regan’s diagnosis (see also Kinder and Houston, 1987). As Williams (2011: 218–19) contends:

The original text presents a psychological diagnosis of hysteria that precedes and challenges the metaphysical explanation of Regan’s behavior which has been accepted culturally due to the enduring popularity and notoriety of the film.

Friedkin’s adaptation does not offer the viable choice presented by the novel. Despite the meticulous attention to scientific accuracy, the possible medico- or psy-scientific explanation for Regan’s behaviour is dismissed, and the film conservatively ‘re-establishes and asserts the patriarchal Christian moral order’ as Regan and indeed 1970s America’s saviour (ibid.: 221).

Science advisors are now common practice in contemporary Hollywood (Kirby, 2008), but it was still relatively unusual for film-makers to consult with a whole team of scientists and medical professionals in the preparation, shooting, and dissemination of their films. The director also worked with a number of technical consultants (both religious and scientific), who were listed together in the film’s end credits and in the press packs: Rev. John Nicola, SJ; Rev. Thomas Bermingham, SJ; Rev. William O’Malley, SJ; Prof. Norman E. Chase, MD; Herbert E. Walker, MD; and Arthur I. Snyder, MD. In personal correspondence with hospitals, research centres, and specific churches, further members of both the scientific and religious communities were recognised for their advice, support, and in some cases on-screen involvement.^23^ The majority of the nurses and doctors seen in the medical sequences were employees of the Goldwater Memorial Hospital, where the scenes were shot.^24^ Again, Friedkin sought precision, this time scientific, to give balance and to underpin the validity of the science in order to allow for faith to be logically positioned as the last hope for salvation.

William Friedkin had built a relationship with the USCCB-OFB, and correspondence between him and the officials at the office were cordial and supportive. As Brother Thomas Allen, a central figure at NCOMP, remarked in his personal review of *The Exorcist* (dated 26 December 1973), ‘The film is strong propaganda for Christ, the Jesuits, and the Catholic Church’, and Allen openly endorsed Blatty and Friedkin’s ‘risky venture’. This review was not released publicly but was included in clergy and lay Catholic reviews amalgamated into a collective Catholic response to the film later published in the *Catholic Film Newsletter*. Internal discussions reflected this attitude and the grudging approval of a film that offered ‘salutary reflections on religious belief and the limits of science’ in spite of its foul language, inappropriate images of child sexuality, and violence (NCOMP, 1974).

## Science, the body, and the Devil

*The Exorcist* signified and solidified the emergence of ‘A-movie’ horror, stretching the limits of a newly liberal Hollywood. Alongside its place within a turn of Catholic-themed narratives, the film also formed part of a subgenre of horror film focussing on medical procedures and the horrors that could be enacted upon the human body in the name of science, including *Rabid* (1977) and *Coma* (1978). In the medical horrors of the 1970s, ‘medical technocracy replaced the Gothic villain and the hospital became the castle’ (Badley, 1995: 24). Increasing trust and reliance on medical technology emerged as a core locus of fear in this nascent subgenre that extrapolated advances in the biosciences in the wake of the 1960s biotech revolution, where ‘fascination mingled with fear’ (Wald, 2012: 188). Scientific innovation and the business of science and medicine became part of the genre and reflected broader considerations of how the male-dominated medical profession viewed and commodified the patient body. *The Exorcist* revelled in ‘body horror and its powers of revulsion’ (Cruz, 2012: 161), and advances in medicine were ‘recast’ as the ‘unknown’ in the place of supernatural spectres or monstrous humans (Boss, 1986: 19).

The on-screen battle between science, God, and the Devil is fought in *The Exorcist* with unprecedented clinical detachment on *and* in a child’s body. As Octavia J. Cade (2016: 67) argues, the archaeologists digging the historical Iraqi site in the film’s prologue are doubled in the later hospital scenes, where ‘Regan’s body is excavated’ by medics sifting through possible somatic causes. Like archaeologists methodically exhuming evidence of past civilisations, medical professionals dig deeper into Regan physically in increasingly invasive and painful procedures. However, it is only once a psychiatrist is able to breach Regan’s subconscious through hypnosis and speak to the ‘demon’ buried inside her that those attempting to diagnose begin to consider possible causes and supernatural solutions. Although it should be noted that the clinical opinion expressed by the hospital doctors – that Regan’s mother should contact an exorcist – is based upon the notion that Regan’s illness is psychological and that the exorcism would be ‘a form of shock treatment’ to relieve the patient of her delusion of demonic possession (ibid.: 68). Doctors must invade and harm the child’s body in their attempts to diagnose her affliction; Regan’s body is not her own as she becomes an object for scientific experimentation.

The demonic bodily invasion means that priests must also harm Regan in order to save her in mind and body. Whereas in the medical sequences Regan loses her bodily autonomy to machines and scientists, in the scenes where demonic possession is identified as the cause, she has lost her body, mind, and morals to the Devil. The incongruence of the obscene tirades and vile actions enacted by an innocent child further underscores the difficulty the scientist-priests have with concluding that an exorcism is required.^25^ There is no concrete medical explanation, and Karras, framed as the religious sceptic, eventually and begrudgingly accepts that the Catholic Church and the ancient rite of exorcism is their only option. His ultimate sacrifice, which results in his death, saves the child from further physical and psychological harm, although Regan, once relieved of her affliction/possession, does not remember the period when the demon emerged as the dominant personality, nor its eventual exorcism. Only the memories of medical testing and bodily experimentation linger.

## Conclusion

In *The Exorcist*, Regan is not the only subject to be probed and morally usurped; the inherent trust in science and its apparent capacity for logic, realism, and healing is also questioned. Where clinical and at times seemingly barbaric science fails, faith and ritual save the possessed child. Medical science neither diagnoses nor cures Regan, but that is not to say that it could not in the future; in the film, figures from both the religious and the scientific community are open to being wrong and hope that science could provide an answer. They both search for alternatives and evidence rather than immediately resorting to worst-case-scenario actions: exorcism and invasive procedures.

Reports of Catholic outrage were a means of promoting *The Exorcist* rather than an accurate reflection of the Catholic Church’s nuanced response to the film and its scientific and religious content. Warner Bros.’ marketing team was, however, eager to exploit reports of fainting fits, demonic possession, and religious outrage to boost sales and news coverage – a strategy that actively contradicted Friedkin’s respectful and collaborative approach.^26^ Yet the Catholic Church did not reject the film and its message – in internal correspondence, letters, and even some centrally released film reviews, the church acknowledged the film’s positive portrayal of the power of faith and the Catholic Church. This encouraging response was not undermined by their rejection of the way this message was framed with obscene language and disturbing sexual imagery.

By 1973, mainline Christian groups had lost their direct influence over the Hollywood film industry but had managed to develop alternative ways of communicating their responses to individual films and responding to the changing societal attitudes those films reflected. Instead of vilifying the industry, the Catholic Church in particular chose to engage with the films being released and hoped to shape the discussions taking place both in and outside their congregation. They placed the onus on and their trust with the audience, who could make their own decisions about what they would see and how they would respond. Many of the science-based films released in the immediate post-censorship era were thought to deify science, with scientists providing salvation through their own sacrifices, most notably in the *Omega Man* (1971), where a scientist’s blood sacrifice saves humanity (Chambers, 2019). *The Exorcist*, however, presents scientists as fallible in a situation where their best efforts cannot explain away the mystery.

Over the decades since the release of *The Exorcist*, the Catholic Church’s reception of the film has transformed, just as attitudes to what is acceptable on screen have also continued to change. The Catholic Church now more openly embraces films that might be seen to contradict or disturb Catholic rites and values, seeing them as an opportunity for discussing faith. For example, early viewing guides produced in the mid 1970s began this trend and included pamphlets on *The Exorcist* alongside a broad range of movies intended as a tool for discussion. The *Exorcist* viewing guides showed an understanding that viewers would interpret the film as fiction and not documentary. For the original audience, *The Exorcist* was shocking in its portrayal of realistic science, sex, and violence (physical and verbal), but it was the beginning rather than the peak of horror imagery. *The Exorcist* became an iconic film not only in cinema history but also in US cultural history and its responses to the psy-sciences and religion. This discussion has closely focussed on *The Exorcist* because it became a metonym for broader discussions of science and religion and the unknown and seemingly uncontrollable evils that emerged in 1970s America. This was a moment in US history when science and religion were critiqued together as fallible institutions despite their apparent incompatibilities – and *The Exorcist* offered a contained cultural space for these valuable discussions to take place. Even as the *The Exorcist* nears its 50th anniversary, it continues to create fear and discomfort for both the viewers and its characters due to what is seen and unseen and known and unknown in both religion *and* science.

## Notes

1. Nat Segaloff, telephone interview with William Friedkin, January 1974, Margaret Herrick Library, Academy of Motion Picture Arts and Sciences, Los Angeles (hereafter ‘Margaret Herrick Library’), Nat Segaloff Research Papers, *The Exorcist* Box 1, no. 8.

2. Raschke, C. (1974, 18 December) ‘Exorcising the Devils of Watergate: Why the Prince of Darkness Is Having His Day’, *The Christian Century*: 1196–8 (1196), microform, Library of Congress, vol. 91, reel 44, *Christian Century* (1900–) collection, BR1.C45.

3. Raschke, ‘Exorcising the Devils of Watergate’, 1198.

4. Letter from Rev. John S. Banahan to William Friedkin, 10 September 1973, Margaret Herrick Library, William Friedkin Papers, 1959-1997, *The Exorcist* correspondence files, file 27. Rev. John S. Banahan was the director of the Archdiocese of Chicago’s Office of Radio and Television (1957–78).

5. Released for researcher consultation in 2015: Margaret Herrick Library, William Friedkin Papers, 1959-1997.

6. The film was given an A-IV classification, which was applied to films that were ‘morally unobjectionable for adults, with reservations’, and ‘while not morally offensive themselves, require caution and some analysis and explanation as a protection to the uninformed against wrong interpretations and false conclusions’ (Skinner, 1993: 153).

7. Prior to 1965, the Legion’s film classification system, intended as a guide for Catholics in deciding whether a movie was appropriate for family viewing, was split into three categories: A (morally acceptable), B (morally objectionable in part), and C (condemned). B and C classifications could seriously affect the box office receipts for a movie, so film-makers were eager to avoid them (Black, 1998). The A classification was then subsequently divided into A-I (suitable for all audiences), A-II (suitable for adults and adolescents), and A-III (suitable for adults only). In 1965, A-IV was added to replace the C rating and was approved for adults only with reservations. In 1978, a new O rating was incorporated to condemn ‘morally offensive’ films.

8. All information in this article on NCOMP’s activities comes from the individual film files in the NCOMP review files: American Catholic History Research Center, Catholic University of America, Washington, DC, Series 1, Review Files, ca.1931-2010, Records of the United States Conference of Catholic Bishops (USCCB) Communications Department/Office of Film and Broadcasting (hereafter ‘USCCB Records’).

9. Further evidence of the pre- and mid-production discussion concerning Friedkin’s use of actual Catholic consecrated venues and liturgy can be found in the USCCB Records. For example, in the *Exorcist* files held in this collection, a letter (8 May 1972) from Rev. Monsignor E. Robert Arthur (chairman, Liturgy Commission, Chancery Office, Archdiocese of Washington, DC) to Rev. Patrick J. Sullivan, SJ (Department of Film and Broadcasting, USCCB NY) noted that they would not allow for ‘a scene of desecration in the church itself’ even though the church ‘appreciates [that] the total impact of the film would seem to be commendable’.

10. The weekly ‘Inside Religion’ column was syndicated across newspapers in Pennsylvania, Maine, and New York state, including the *Pittsburgh Post-Gazette*, the *Pittsburgh Press*, the *Palm Beach Post*, the *Sumter Daily Item*, the *Erie Daily Times*, and the *Plain Dealer*. See Library of Congress Microfilm Collection, Washington, DC.

11. A phone call recorded between Warner Bros. publicists and the manager of Town and Country Cinema (Columbus, OH), Gary Foulkes, showed the impact that boycotts and flyering had, with Foulkes remarking that ‘we’re getting prodded by local churches, they’re passing out anti-Exorcist material, which is really helping more than anything else…They’re using the scare factor as a deterrent…it’s the thing that’s drawing them the most’ (ellipses in original). Telephone call with Gary Foulkes, 27 February 1974, Margaret Herrick Library, William Friedkin Papers, 1959-1997, *The Exorcist* Exhibition, Folder 36.

12. P. B. Ross, ‘Memo: Friedkin’, 1973. Collection of documents that follow a Warner Bros. memo: ‘“Exorcist” – Material for Bill Friedkin’s Book’, Margaret Herrick Library, William Friedkin Papers, 1959-1997, ‘Background Material on “The Exorcist”’, *The Exorcist* Publicity Files – Stories, File 65.

13. Ibid.: 7–8.

14. Ibid.: 7–8.

15. Although based in London, *New Scientist* was and continues to be widely distributed in the United States and Australia.

16. Nat Segaloff, interview with William Peter Blatty (annotated transcript – Blatty notes), 10 April 1988, Margaret Herrick Library, Nat Segaloff Research Papers, *The Exorcist* Box 1, no. 8.

17. Ibid.: 10 (emphasis in original).

18. The hospital sequences with Karras’ dying mother also highlight the horrific nature of a medical system that punishes the poor – even when, like Father Karras, their ‘vow of poverty’ is grounded in devotion to God and His congregation, it has ‘brutal’ consequences on the treatment and later death of his ailing parent (Phillips, 2005: 115–16).

19. The first static magnetic resonance (nuclear MR) images appeared in *Nature* in March 1973 (Lauterbur, 1973), as the film was in production. Computed tomographic (CT) scans, which replaced the procedures shown and suggested in the film, were not installed into hospitals until 1973, after film production had wrapped (Hoeffner *et al.*, 2012).

20. These notes and hand-drawn and annotated diagrams are not available for reproduction but can be seen in Margaret Herrick Library, William Friedkin Papers, 1959-1997.

21. Friedkin, quoted in Ross, ‘Memo: Friedkin’, p. 7. See also Margaret Herrick Library, William Friedkin Papers, 1959-1997, *The Exorcist* Correspondence File, Folder 27.

22. As with Blatty’s novel, the film focussed specifically on the Jesuits (the Society of Jesus), the largest male religious order within the Roman Catholic Church, devoted to education, the mission, and charitable works.

23. Correspondence between Friedkin and actor Max von Sydow made reference to set visits from Fr Nicola to check that everything was ‘technically correct’ – he praised checking on Catholic detail in the same way as he did medical terms and imagery. Letter from William Friedkin to Max von Sydow, 23 January 1973, Margaret Herrick Library, William Friedkin Papers, 1959-1997, *The Exorcist* Script-Exorcism, Folder 74.

24. One of the radiology technicians, Paul Bateson, was convicted for the murder of *Variety* theatre critic Addison Verrill, and was known to the New York City tabloid press as ‘the Trashbag Killer’. He confessed to multiple murders (although he was not charged) and partially inspired Friedkin’s 1980 film *Cruising*, about a serial killer targeting gay men; Bateson was an uncredited consultant for the film (see Friedkin, 2013: 358–61).

25. Merrin is an archaeologist and Karras is a psychiatrist; one physically digs for the evidence and the other attempts to resolve problems that are psychologically buried beneath the surface.

26. See the inter-office memo between Warner Bros. publicists attached to an article clipping from the *Los Angeles Times* article about extreme responses to *The Exorcist* (the article in question is Goodman, 1974). The note said: ‘Be advised that this type of story and coverage is possible and all possible efforts should be expended to get this type of coverage in each city’. Inter-office Warner Bros. memo from Leo Wilder to Marty Weiser, subject: *The Exorcist*, 8 January 1974, Margaret Herrick Library, Marty Weiser Papers, *The Exorcist*, Folder 184.
